# Determination of the frequency, species distribution and antimicrobial resistance of staphylococci isolated from dogs and their owners in Trinidad

**DOI:** 10.1371/journal.pone.0254048

**Published:** 2021-07-02

**Authors:** Sharianne Suepaul, Karla Georges, Chandrashekhar Unakal, Filip Boyen, Jamie Sookhoo, Khalil Ashraph, Anisah Yusuf, Patrick Butaye

**Affiliations:** 1 Department of Basic Veterinary Sciences, School of Veterinary Medicine, Faculty of Medical Sciences, The University of the West Indies, St. Augustine, Trinidad and Tobago; 2 Department of Paraclinical Sciences, School of Medicine, Faculty of Medical Sciences, The University of the West Indies, St. Augustine, Trinidad and Tobago; 3 Department of Pathology, Bacteriology and Avian Diseases, Faculty of Veterinary Medicine, Ghent University, Ghent, Belgium; 4 School of Veterinary Medicine, Ross University, Basseterre, St. Kitts and Nevis; The Rockefeller University, UNITED STATES

## Abstract

The close contact between humans and their dogs can lead to the commingling of staphylococci and the exchange of mobile genetic elements encoding antimicrobial resistance. The objectives of this study were to determine the species distribution and antimicrobial resistance patterns of staphylococci colonizing canine pets and their owners in Trinidad. Staphylococci were isolated from canine pets and their owners and identified using MALDI-TOF mass spectrometry. Antimicrobial susceptibilities were determined using the Kirby-Bauer disc diffusion method against seven classes of antimicrobial agents. A total of 440 staphylococci were isolated from 112 canine pets and their owners, 53.4% were from canine pets and 46.6% were from owners. Twenty-four species were detected, of which, most isolates (32.5%) belonged to the *Staphylococcus intermedius* group (SIG). *S*. *sciuri* was the most common species of coagulase-negative staphylococci (CoNS) comprising 22.3% of all isolates. Antimicrobial resistance was highest against commonly used antimicrobials, such as penicillin (51.4%), tetracycline (26.1%) and trimethoprim/sulfamethoxazole (18.6%). These antimicrobials also comprised the most common multidrug resistance (MDR) combination. Overall, 19.1% of isolates displayed multidrug resistance. No methicillin-resistant *Staphylococcus aureus* (MRSA) isolates were detected. However, methicillin resistance was detected in 13.3% and 15.1% of coagulase-positive staphylococci (CoPS) and the CoNS+CoVS (combined CoNS and coagulase-variable staphylococci) group respectively. The presence of methicillin-resistant staphylococci is worrisome because there is the potential for the transfer of these strains between dogs and humans. These strains may act as a reservoir of resistance genes.

## Introduction

Staphylococci are generally commensals of the skin and mucous membrane of the upper respiratory tract of both humans and animals [1, 2]. These organisms can also colonize the digestive tract [3]. Staphylococci can also be found in the environment and are adapted to survive under adverse conditions [4].

To date, there are at least 71 recognized species and 30 subspecies of staphylococci [5]. Historically, staphylococci have been divided into two main groups, coagulase-positive staphylococci (CoPS) and coagulase-negative staphylococci (CoNS). Initially, coagulase positivity was associated with virulence as coagulase production promotes the clotting of blood, protecting the organism from the host’s immune defenses [6]. CoNS were considered insignificant in causing infections and were thought to be contaminants [7].

Presently, staphylococci can be divided into 15 cluster groups and six species groups based on 16s rRNA sequences [8]. In humans, the most clinically important species is *S*. *aureus*, which is responsible for various infections including soft tissue infections [9] and pneumonia [10]. In dogs, the most important species is *S*. *pseudintermedius* [11] which causes mainly otitis and skin infections [12, 13].

Lately, there is increasing data on the importance of CoNS in causing infections. This is exemplified by the multitude of virulence factors associated with these organisms [14]. CoNS are considered to be a major cause of nosocomial infections in humans with *S*. *epidermidis* [7] and *S*. *haemolyticus* [15] being major species involved in these infections. CoNS such as *S*. *chromogenes* and *S*. *haemolyticus* have been implicated in causing mastitis in cows [16], while species such as *S*. *schleiferi* have been found to cause otitis and skin infections in dogs [17].

Since the 1960’s, there has been a global increase in infections involving methicillin-resistant staphylococci, more specifically methicillin-resistant *Staphylococcus aureus* (MRSA) in humans [18] and animals [19]. MRSA infections are classified into three main categories, healthcare associated-MRSA (HA-MRSA), community associated-MRSA (CA-MRSA) [20] and livestock associated-MRSA (LA-MRSA) [21]. There may be specific staphylococcal chromosomal cassette *mec* (SCC*mec*) types associated with these different categories of MRSA. SCC*mec* types IV and V are often associated with CA-MRSA and SCC*mec* types I, II and III are associated with HA-MRSA [22]. However, in the Caribbean the major circulating strains in both hospitals and the community include SCC*mec* I, II, III and the USA300 strain [23–25] which usually carries SCC*mec* type IV. The main SCC*mec* types detected in LA-MRSA are SCC*mec* IVa and V [21, 26]. LA-MRSA, while considered of low pathogenicity, is capable of colonizing many different animal species including humans. LA-MRSA has been reported at low prevalence rates inclusive of 0.9 to 2.1% of farmed pigs [27, 28], 2.3% of abattoir workers [28] and 0.7% of broilers [29] tested in Trinidad. Typically, HA-MRSA and LA-MRSA strains are resistant to multiple antibiotics, while CA-MRSA strains are frequently less resistant. However, emerging data suggests that this may no longer be the case due to the global transmission of MDR CA-MRSA strains [30].

From the end of the 1990’s onwards, there were many reports on the increasing prevalence of methicillin-resistant *Staphylococcus pseudintermedius* (MRSP) in dogs [31]. Several strains of *S*. *pseudintermedius* have acquired methicillin resistance [32]. Typically, these strains are also resistant to multiple classes of antimicrobial agents and likewise are capable of causing infections which are difficult to treat [33]. MRSA and MRSP have been detected in both humans and dogs, though MRSA may only temporarily colonize dogs. Dogs may be involved in the transmission of MRSA amongst humans [34].

While there are numerous reports on the prevalence of antimicrobial resistance in CoPS, less is known about the other staphylococcal species. In CoNS, methicillin-resistance has been frequently reported, at times at higher prevalence values than for CoPS [35]. Resistance in CoNS can be of importance as they may act as a reservoir of resistance genes [36].

In light of the potential exchange of different staphylococcal species between humans and animals, there are two main objectives of this study: first, to identify staphylococci (CoPS, CoNS and CoVS) present at chosen sites in dogs and their owners and second, to determine and compare the antimicrobial resistance patterns of the two sample populations.

## Materials and methods

### Study area, population and sampling strategy

Samples were collected at 11 veterinary clinics throughout the island of Trinidad, between October 2016 to October 2017. Two clinics located in the north, two in the south, two in the east, three in the west and two in central Trinidad were sampled. The Veterinary Hospital at the Eric Williams Medical Sciences Complex, Mt. Hope, was also included in this study. Samples were taken from apparently healthy dogs during their visit to these clinics for health checks or vaccinations. Swabs (Copan Transystem® culture swab transport system, Brescia, Italy) were used to collect samples from the mouth, nares and palms of dog owners and from the mouth, nares, and ventral abdomen of their dogs. Samples were transported to the laboratory and processed the same day.

### Isolation and identification of staphylococci

All of the media used in this study was manufactured by Oxoid, Hants, United Kingdom (UK). Each swab was transferred to 10 mL buffered peptone water (BPW) and incubated for 24 h at 37°C. After incubation, tubes were vortexed and 1 mL of this culture was transferred to 9 mL of brain-heart infusion (BHI) broth and incubated aerobically overnight at 37°C. One loop-full of this culture was then plated onto freshly prepared mannitol salt agar (MSA) to isolate presumptive staphylococci. These MSA plates were incubated for 24 to 48 h at 37°C.

Plates were observed for growth and one colony of each observed morphology was selected (mannitol fermenting and non-mannitol fermenting) and purified on 5% sheep blood agar plates, incubated for 24 h at 37°C. Colony morphologies and haemolytic activities of these presumptive *Staphylococcus* spp. were recorded and the coagulase test (both slide and tube tests) were performed. To avoid duplicates, isolates from each sample were considered unique if any of the observed characteristics (colony morphology, haemolysis on blood agar, coagulase activity or mannitol fermentation) were different. Staphylococci were identified using matrix-assisted laser desorption/ionization time-of-flight (MALDI-TOF) mass spectrometry analysis (MALDI Biotyper, Bruker) following the manufacturer’s instructions and as described previously [37, 38]. Only results with score values ≥1.700 were considered for this study. Although manufacturer’s cutpoint for species-level identification was described as 2.000, previous reports have indicated that a reduction of the species level cutpoint to ≥1.700 improved the species-level identification rates (from 64 to 92% for the existing commercial database) for CoNS [37]. Hence, a cutpoint of ≥1.700 was utilized in this study.

### Antimicrobial susceptibility testing

Antimicrobial susceptibility was determined using the Kirby-Bauer disc diffusion method according to the European Committee on Antimicrobial Susceptibility Testing (EUCAST) guidelines [39]. Briefly, fresh suspensions of pure isolates were prepared to the density of 0.5 McFarland turbidity standard and Mueller Hinton II agar plates (Oxoid, Hants, United Kingdom) were inoculated. Six classes of antimicrobial agents as well as cefoxitin and oxacillin discs (for methicillin resistance screening) were used. All discs were produced by Oxoid, Hants, United Kingdom. Positive and negative controls were used in screening for methicillin resistance. Plates were incubated aerobically at 35 ± 1°C for 16–20 h and zone diameters were measured manually using an Antibiotic Zone Scale-C™. EUCAST clinical breakpoint tables [40] were observed for these samples to differentiate between susceptible and resistant isolates (S1 Table).

### Statistical analyses

The data was analyzed in two ways, firstly, in terms of frequency of isolation in which percentages were calculated using the total number of *Staphylococcus* isolates obtained in a specified category as the denominator. Secondly, prevalence values were calculated using the total number of humans (n = 112) or dogs (n = 112) as the denominator. The prevalence values indicate the percentage of humans or dogs which were culture positive for one or more of a specified *Staphylococcus* spp. or group. Prevalence values may not sum to 100% because some humans and dogs were host to more than one species.

The data was analyzed using Statistical Package for Social Sciences (SPSS Version 23, IBM Corp., Armonk, NY). The Pearson’s chi-square test with Yates’ continuity correction and the two-sided Fisher’s exact test (for 2 × 2 contingency tables with expected frequencies of <5) were used for comparisons between categorical variables to determine whether there were associations between (i) the frequency of isolation of *Staphylococcus* spp. and the host species (humans or dogs), (ii) the frequency of resistance to antimicrobial agents and the host species (humans or dogs) and (iii) the prevalence of the SIG in dogs and humans (for which the odds ratio was also calculated). Associations between the variables were deduced with 95% confidence intervals (CIs) and *P*-values < .05 were considered significant.

### Ethical approval

Ethical approval was obtained from the University of the West Indies Ethics Committee, reference number: CEC227/06/16. Witnessed verbal informed consent was obtained from all owners for sample collection. No minors were sampled during this study.

## Results

### Distribution of *Staphylococcus* spp. amongst humans and dogs

#### Overall distribution of *Staphylococcus* spp

Of 112 canine owners and their dogs, 89.3% of owners and 87.5% of pets were positive for one or more *Staphylococcus* species. Overall, 440 unique *Staphylococcus* isolates were obtained, 53.4% of these isolates originated from canine pets and 46.6% originated from their owners (S1 Fig). A total 24 different species of *Staphylococcus* were identified (S2 Table). However, staphylococci belonging to the *Staphylococcus intermedius* group (SIG) including *S*. *pseudintermedius*, *S*. *intermedius* and *S*. *delphini* were categorized together. Species of the SIG were the most frequently detected, comprising 32.5% (143/440) of all isolates, followed by *S*. *sciuri* comprising 22.3% (98/440) of all isolates. Most of the isolates (52.3%) were identified as CoNS, 41.1% of isolates were CoPS and 6.6% were CoVS.

#### Distribution of *Staphylococcus* spp. in humans

A total of 205 *Staphylococcus* isolates were cultured from humans, 59 (28.8%) from the mouth, 62 (30.2%) from the nares and 84 (41.0%) from the palms (S1 Fig). Most of the isolates from humans (54.6%) were CoNS, 40.5% were CoPS and 4.9% were CoVS.

**Fig 1:**
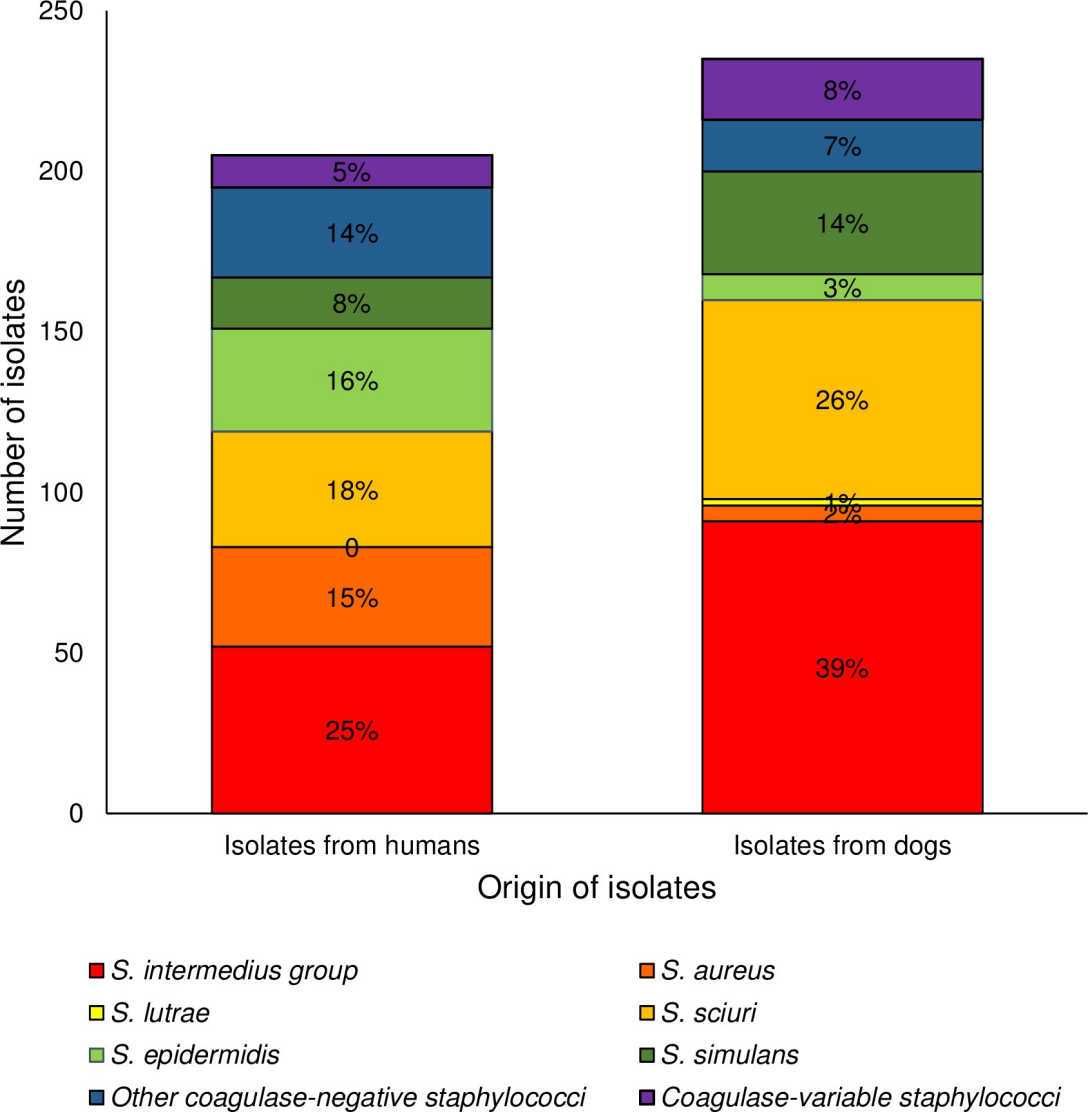
Frequency of isolation of *Staphylococcus* spp. from humans (n = 205) and dogs (n = 235).

Commonly isolated CoPS in humans included members of the SIG at 25.4% and *S*. *aureus* at 15.1% (Fig 1). The frequency of detection of the SIG was significantly higher (*P* < .05) on the palms than other sample sites, with 82.9% (29/35) of CoPS isolates found in this location belonging to the SIG. Commonly isolated CoNS on humans were *S*. *sciuri* (17.6%), *S*. *epidermidis* (15.6%) and *S*. *simulans* (7.8%).

Overall, 51.8%, 62.5% and 8% of humans (n = 112) were culture-positive for one or more isolates of CoPS, CoNS and CoVS respectively. Members of the SIG and *S*. *aureus* were the most abundant CoPS, detected on 40.2% and 18.8% of humans respectively. The three major species of CoNS were *S*. *sciuri*, *S*. *epidermidis* and *S*. *simulans*, detected at a prevalence of 26.8%, 23.2% and 10.7% respectively. Only two species of CoVS were present on humans, *S*. *schleiferi* and *S*. *agnetis* at a prevalence of 7.1% and 0.9% respectively (S2 Fig).

#### Distribution of *Staphylococcus* spp. in canine pets

A total of 235 *Staphylococcus* isolates were cultured from pet canines, 77 (32.8%) from the mouth, 88 (37.4%) from the nares and 70 (29.8%) from the abdominal region (S1 Fig). The frequency of isolation of staphylococci was similar for all three sample sites. Most of the isolates originating from pet canines were CoNS, (50.2%), 41.7% were CoPS and 8.1% were CoVS.

Commonly isolated CoPS in dogs consisted of the SIG (38.7%) followed by *S*. *aureus* (2.1%) and *S*. *lutrae* (0.9%) (Fig 1). Commonly isolated CoNS in pet canines were *S*. *sciuri* and *S*. *simulans* comprising 26.4% and 13.6% of all canine isolates respectively.

Overall, 48.2%, 62.5% and 10.7% of canine pets (n = 112) were culture-positive for one or more isolates of CoPS, CoNS and CoVS respectively. Members of the SIG, *S*. *aureus* and *S*. *lutrae* were cultured from 45.5%, 4.5% and 1.8% of dogs respectively. CoNS including *S*. *sciuri*, *S*. *epidermidis* and *S*. *simulans* were cultured from 38.4%, 6.3% and 22.3% of dogs respectively (S2 Fig). Only two species of CoVS were present on dogs, *S*. *schleiferi* and *S*. *agnetis* which were cultured from 9.8% and 1.8% of dogs respectively.

#### Comparison of the distribution of *Staphylococcus* spp. in canine pets and humans

Statistically significant associations were observed between the frequency of isolation of some of the *Staphylococcus* spp. and the host species (humans versus dogs). The frequency of isolation of the SIG, *S*. *sciuri*, and *S*. *simulans* was higher in dogs than in humans (*P*-values < .05). While, the frequency of isolation of *S*. *aureus* and *S*. *epidermidis* was higher in humans than in dogs (*P*-values < .05). *S*. *equorum*, *S*. *xylosus*, *S*. *cohnii* and *S*. *saprophyticus* were only isolated from canines, while *S*. *hominis*, *S*. *caprae*, *S*. *pasteuri* and *S*. *condimanti* were only isolated from humans in this study (S2 Table).

A total of 45 humans were SIG-positive, of these 27 (60.0%) also had dogs which were SIG-positive (Table 1). Additionally, of the 67 humans who were SIG-negative, 43 (64.2%) had dogs which were SIG-negative. There was a significant association between the prevalence of the SIG in dogs and their human counterparts in this study (*P*-value <0.05). The odds ratio (OR) analysis indicates that humans who owned dogs which were SIG-positive displayed higher odds of being SIG-positive than humans who owned a SIG-negative dog (OR 2.69, 95% CI 1.23–5.85).

**Table 1:** 2x2 contingency table used to perform the Pearson’s chi-square test with Yates’ continuity correction and odds ratio on the SIG prevalence data in humans and dogs.

Matched pairs		Humans		
		SIG-positive	SIG-negative	Total
Dogs	SIG-positive	27	24	51 (45.5%)
	SIG-negative	18	43	61 (54.5%)
	Total	45 (40.2%)	67 (59.8%)	112 (100%)

### Antimicrobial resistance patterns

#### Overall antimicrobial resistance patterns

Half of the staphylococci isolated in this study (n = 440) were resistant to penicillin (51.4%). Lower resistance values were observed against tetracycline (26.1%), trimethoprim/sufamethoxazole (18.6%), ciprofloxacin (18.2%), amikacin (15.7%), and chloramphenicol (4.3%). Overall 14.3% of isolates screened positive for methicillin resistance (MR).

#### Antimicrobial resistance of CoPS

The CoPS group consisted of 181 isolates, 45.9% from humans and 54.1% from dogs. Overall for the CoPS the frequency of resistance was highest to penicillin (63%), followed by tetracycline (34.3%), trimethoprim/sulphamethoxazole (32.6%), ciprofloxacin (16.6%), chloramphenicol (8.3%) and amikacin (3.9%).

**Fig 2:**
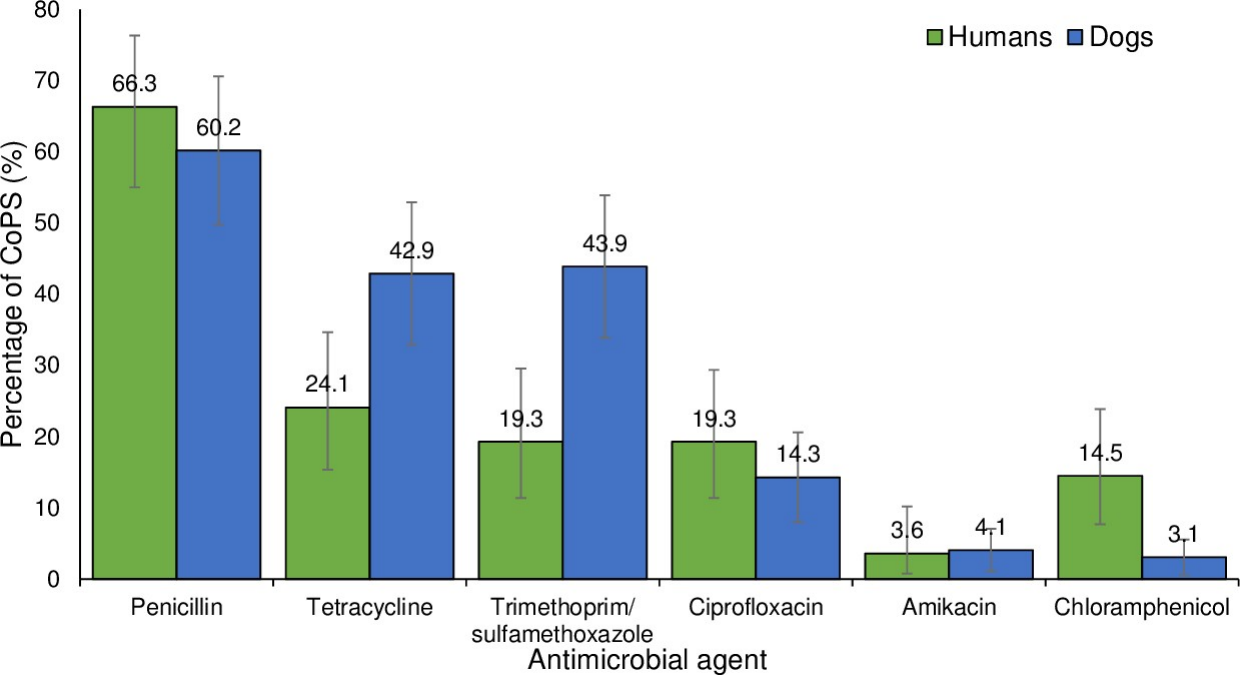
Frequency of detection of antimicrobial resistance in coagulase-positive staphylococci (CoPS) isolated from humans (n = 83) and dogs (n = 98).

Amongst the CoPS isolated from humans (n = 83), the frequency of resistance was highest to penicillin (66.3%) followed by tetracycline (24.1%), trimethoprim/sulfamethoxazole (19.3%), ciprofloxacin (19.3%), chloramphenicol (14.5%) and amikacin (3.6%) (Fig 2). None of the 31 *S*. *aureus* isolates cultured from humans were resistant to cefoxitin used for MR screening. However, 9.6% (5/52) of the SIG isolated from humans were MR-positive (when screened using oxacillin) (S3 Table).

Of the 112 humans sampled, 38.4%, 17%, 14.3%, 13.4%, 2.7% and 10.7% were positive for CoPS resistant to penicillin, tetracycline, trimethoprim/sulfamethoxazole, ciprofloxacin, amikacin and chloramphenicol respectively (S3 Fig). The prevalence of MRSP in humans was 4.5% (S4 Table).

Amongst the CoPS isolated from dogs (n = 98), the frequency of resistance was highest to penicillin (60.2%) followed by tetracycline (42.9%) and trimethoprim/sulfamethoxazole (43.9%) (Fig 2). None of the five *S*. *aureus* isolates obtained from dogs were resistant to cefoxitin used for MR screening, however 20.9% of the SIG from dogs screened positive for MR (were resistant to oxacillin) (S3 Table).

Of the 112 dogs sampled, 32.1%, 23.2%, 21.4%, 9.8%, 3.6% and 2.7% were positive for CoPS resistant to penicillin, tetracycline, trimethoprim/sulfamethoxazole, ciprofloxacin, amikacin and chloramphenicol respectively (S3 Fig). The prevalence of MRSP in dogs was 12.5%.

For CoPS, the frequency of resistance to tetracycline and trimethoprim/sufamethoxazole was higher in dogs (42.9% and 43.9% respectively) when compared to humans (24.1% and 19.3% respectively) (P < .05). However, the frequency of resistance to chloramphenicol was higher in humans (14.5%) when compared to dogs (3.1%) (P < .05).

#### Antimicrobial resistance of CoNS and CoVS

CoNS and CoVS were combined for the analyses to produce the CoNS+CoVS group, which consisted of a total of 259 isolates, 47.1% from humans and 52.9% from dogs. Overall for this group, the frequency resistance to penicillin, amikacin, tetracycline, ciprofloxacin, trimethoprim/sulfamethoxazole and chloramphenicol was 43.2%, 24%, 20.5%, 19.3%, 8.9% and 1.5% respectively.

**Fig 3:**
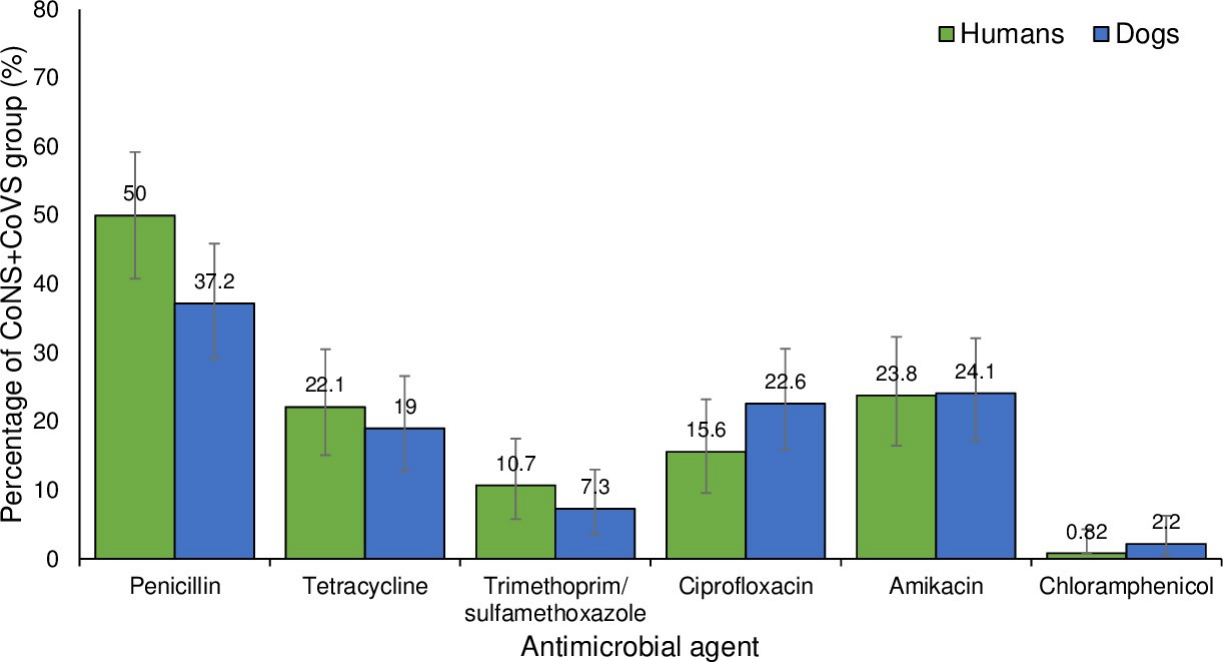
Frequency of detection of antimicrobial resistance in the CoNS+CoVS group isolated from humans (n = 122) and canine pets (n = 137).

Amongst the of the CoNS+CoVS group isolated from humans (n = 122), the frequency of resistance was highest to penicillin (50%) followed by amikacin (23.8%), tetracycline (22.1%), ciprofloxacin (15.6%), trimethoprim/sulfamethoxazole (10.7%) and chloramphenicol (0.8%) (Fig 3).

In humans (n = 112), the prevalence of CoNS+CoVS group resistant to penicillin, tetracycline, trimethoprim/sulfamethoxazole, ciprofloxacin, amikacin and chloramphenicol was 42.8%, 20.5%, 9.8%, 16.1%, 22.3% and 0.9% respectively (S4 Fig).

Amongst the of the CoNS+CoVS group isolated from canines (n = 137) the frequency of resistance was highest against penicillin (37.2%), followed by amikacin (24.1%), tetracycline (19%), ciprofloxacin (22.6%), trimethoprim/sulfamethoxazole (7.3%) and chloramphenicol (2.2%) (Fig 3).

In dogs (n = 112), the prevalence of species of the CoNS+CoVS group resistant to penicillin, tetracycline, trimethoprim/sulfamethoxazole, ciprofloxacin, amikacin and chloramphenicol was 34.8%, 21.4%, 7.1%, 18.8%, 22.3% and 2.7% respectively (S4 Fig). For the CoNS+CoVS group, no significant associations were observed between the frequency of resistance to antimicrobial agents and the host species.

MR screening using cefoxitin indicated that 15.1% of isolates of the CoNS+CoVS group (n = 259) were resistant to cefoxitin. Of these 13.1% of CoNS+CoVS canine isolates (n = 137) were resistant to cefoxitin consisting of 12 *S*. *sciuri*, one *S*. *epidermidis*, two *S*. *simulans*, two *S*. *schleiferi* and one *S*. *cohnii* isolate. In humans (n = 122), 17.2% of CoNS+CoVS group screened positive for MR, including six *S*. *sciuri*, five *S*. *epidermidis*, two *S*. *simulans*, five *S*. *haemolyticus*, one *S*. *warneri*, one *S*. *hominis* and one *S*. *schleiferi* isolate. The prevalence of the MR-CoNS+CoVS group was 16.1% in humans and 13.4% in dogs (S4 Table).

Overall, the frequency of resistance to penicillin, tetracycline, chloramphenicol and trimethoprim/sulphamethoxazole was higher in CoPS when compared to the CoNS+CoVS group (*P* < .05). While the frequency of resistance to amikacin was higher in the CoNS+CoVS group compared to the CoPS (P < .05).

#### Multidrug resistance

**Fig 4:**
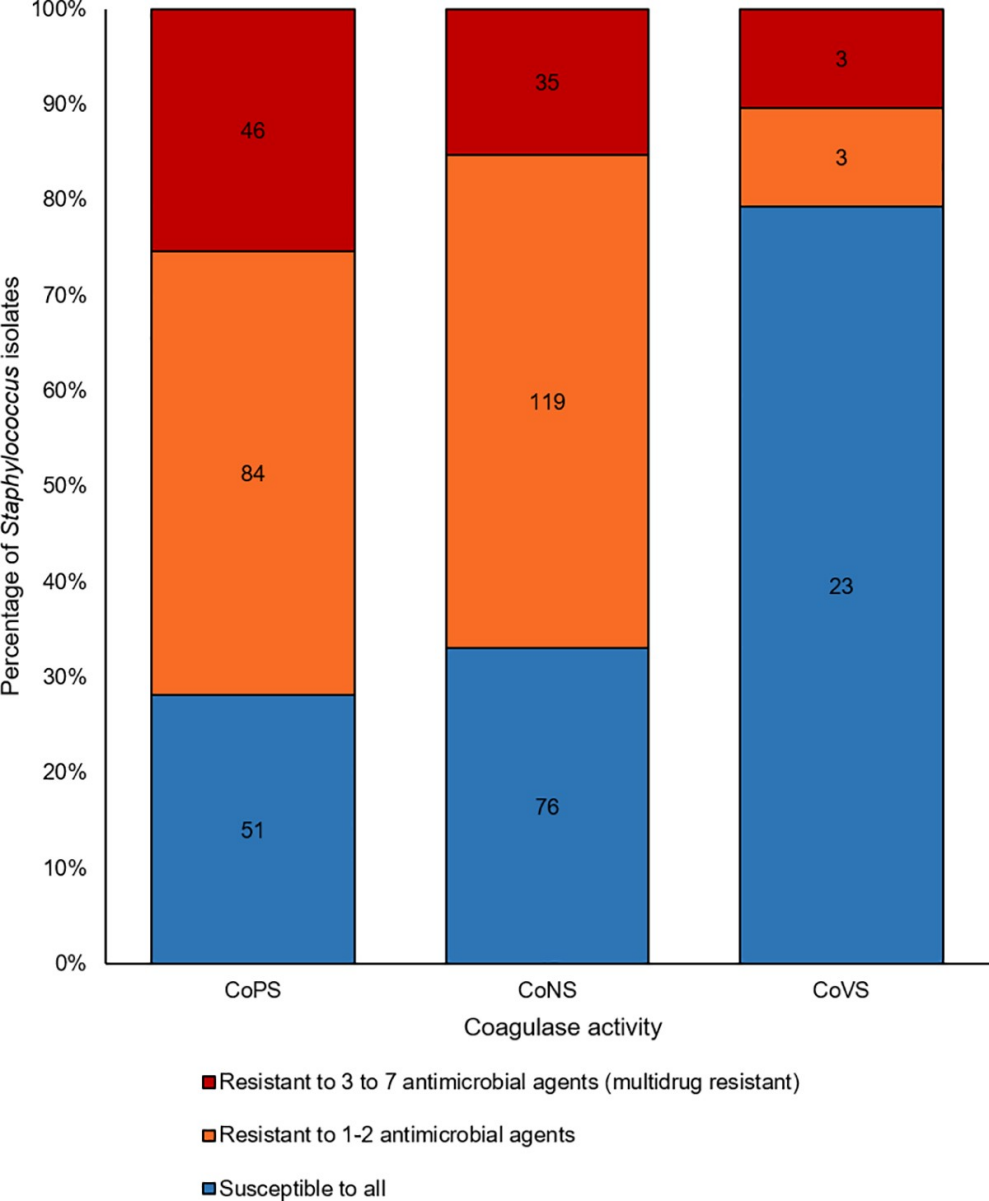
Proportions of multidrug resistant staphylococci.

A total of 71.8% of the CoPS, 67.0% of the CoNS and 20.7% of the CoVS isolates were resistant to 1 or more classes of antibiotics. Multidrug resistant isolates, defined as resistance to three or more antimicrobial classes, were highest in CoPS 25.4% (Fig 4). CoNS and CoVS isolates displayed lower frequency values for multidrug resistance at 15.2% and 10.3% respectively. Overall, 57.1% of MDR isolates were resistant to penicillin, tetracycline and trimethoprim/sulfamethoxozole or combinations which included these antimicrobials. There is a significant association between cefoxitin resistance and multidrug resistance (*P* < .05).

MDR CoPS were detected on 15.2% owners and 17.8% of dogs. MDR CoNS+CoVS group isolates, were detected at a lower prevalence than MDR CoPS in both humans and animals. MDR CoNS and MDR CoVS were detected in 14.2% and 0.9% of humans respectively and in 13.4% and 1.8% of dogs respectively.

## Discussion

This is the first study to report on the species diversity and distribution of CoPS and CoNS present on healthy dogs and their owners in Trinidad and Tobago. A multiplicity of *Staphylococcus* spp. was observed in dogs and humans, most of which have the potential to cause clinical infections. *Staphylococcus* spp. commonly implicated in infections such as *S*. *aureus*, members of the SIG and *S*. *epidermidis* were isolated in this study. Other emerging pathogenic species such as *S*. *sciuri*, *S*. *simulans*, *S*. *haemolyticus*, and *S*. *schleiferi* were also identified.

Overall, the frequency of isolation and prevalence values of CoPS and CoNS were similar in humans and dogs. Interestingly, the predominant *Staphylococcus* spp. isolated in this study were of the SIG and *S*. *sciuri* in both humans and dogs. Even though it has been described that MALDI-TOF MS can distinguish between the species of the SIG, this is still somewhat controversial and it was therefore chosen to group *S*. *intermedius*, *S*. *pseudintermedius* and *S*. *delphini* results, thus reporting these as the SIG [41–44]. However, it can be inferred that most of the canine isolates of the SIG would be *S*. *pseudintermedius* as it is typically associated with dogs [45]. *S*. *intermedius* is mainly associated with pigeons [46], while *S*. *delphini* has been isolated from dolphins [47], penguins [48], horses [49], mink, foxes, badgers and ferrets [50]. Further testing is necessary to differentiate the species of the SIG group.

The predominance of the SIG in dogs is expected. Studies on companion dogs and their owners report prevalence values for *S*. *pseudintermedius* from 13.9% [51] to as high as 87.4% in dogs [52], whereas prevalence values of 4.1% [53] and 5.6% [51] were detected in the nares of humans with companion dogs. We detected a higher prevalence of 40.2% for the SIG in humans. This high occurrence of the SIG in humans may be due to the fact that this study sampled three locations, the palms, nares and mouths in humans, while most published studies investigated the occurrence of the SIG in the nares only [54, 55]. Of all the locations sampled in humans, staphylococci were most frequently isolated from the palms, where the most prominent species were of the SIG.

Furthermore, this study indicates that owners of dogs which were SIG-positive were 2.69 times more likely to be culture-positive for the SIG than humans who owned a SIG-negative dog. This phenomenon could be attributed to the fact that humans have contact with their pets by touching and handling them, thus reflecting the transfer of the SIG from dogs to humans. However, further genomic investigations are necessary to unambiguously demonstrate the exchange of the SIG from dogs to humans.

Additionally, an assessment of the relationship between the SIG and their hosts should be undertaken to determine whether these staphylococci occupy this niche permanently, transiently or are merely transferred during the handling of pet dogs. There is some evidence indicating that persistent carriage of *S*. *pseudintermedius* in humans can occur [54] while others indicate that prolonged colonization with MRSP is rare in humans [56].

Another very important CoPS is *S*. *aureus*, which was detected in 18.8% of humans and 4.5% of dogs. Previous studies have shown that about 30% of healthy humans are colonized with *S*. *aureus*, with 10–20% of human nares being permanently colonized, while a third to half of humans are intermittently colonized and the remainder are never colonized [57–59]. Dogs are normally not colonized with *S*. *aureus*, however the temporary carriage of human *S*. *aureus* strains, especially for those of MRSA is possible [60, 61]. Further genomic investigations are necessary to unambiguously prove the exchange of *S*. *aureus* between humans and dogs in this study.

Approximately half of the isolates obtained in this study were CoNS and the main CoNS species isolated was *S*. *sciuri*. This species has been shown to be abundantly present in dogs [62]. The presence of *S*. *sciuri* is of concern because it is a known reservoir of virulence and resistance genes. These genes can be transferred to other *Staphylococcus* spp. occupying the same habitat [36].

*S*. *epidermidis* is a clinically important CoNS [63] which has been isolated from humans [64], dogs [65] and even pig farms [66]. A high prevalence of *S*. *epidermidis* was expected because this species is considered to be a part of the normal flora in humans. *S*. *epidermidis* has also been regarded as a reservoir of antimicrobial resistance genes [67].

Overall, the antimicrobial resistance patterns of the staphylococci from humans and dogs were similar. There are two possibilities for this observation. One possibility is the potential exchange of these staphylococci between humans and animals living together [60]. Another possibility is that the resistance patterns observed in dogs and owners may be influenced by the use of similar antibiotics.

Resistance to tetracycline and trimethoprim/sulfamethoxazole was higher in CoPS isolated from dogs than humans. These antimicrobials are commonly used in veterinary medicine. It is possible that the CoPS developed resistance to these antimicrobials over time. Although chloramphenicol is not used in humans and animals in Trinidad anymore, it was included in the antimicrobial panel used in this study to assess whether there was any residual resistance. Surprisingly, a higher frequency of chloramphenicol resistance was observed in staphylococci isolated from humans, despite the fact that it is no longer used.

Overall, for the combined group CoNS and CoVS, the frequency of isolation of resistant staphylococci was significantly lower than that of CoPS for penicillin, tetracycline, chloramphenicol and trimethoprim/sufamethoxazole. A low frequency of methicillin-resistance was observed in CoNS. This was unexpected as several reports highlighted the high prevalence of resistance in CoNS and it is suggested that these are reservoirs of resistance genes [68]. However, this study was performed on apparently healthy dogs and humans, none of these were from clinical cases. Hence, the isolates which were obtained were not subjected to selection pressures by antimicrobial agents at the point of sampling, unlike those isolates which may be found in a hospital setting. Additionally, the variations in the frequency of resistance between the CoPS and the CoNS+CoVS group in this study may indicate the importance of developing different zone diameter clinical breakpoints for CoPS, CoNS and possibly even for different *Staphylococcus* species.

Resistance to penicillin was observed as a proxy for determining beta-lactamase activity produced by the *blaZ* gene. Resistance to cefoxitin and oxacillin was observed as a proxy of methicillin resistance (resulting from the presence of the *mecA* gene). Albeit, it is acknowledged that this resistance needs to be confirmed by the molecular detection of the *mecA* gene, because of the many problems associated with the phenotypic determination of this type of resistance. Using the disc diffusion method, no MRSA were observed amongst the isolates from pet canines or their owners. This finding is in agreement with the fact that MRSA colonization in the community in Trinidad and Tobago [69] and other countries [70] is low. Additionally, studies investigating MRSA in households indicates that pets in households where owners were positive were more likely to be MRSA-positive [71]. Also, studies have indicated that MRSA strains detected in household dogs were of human origin and matched that of their owners [70, 72].

Oxacillin-resistant isolates of the SIG were detected in 4.5% of humans and 12.5% of dogs, indicative of methicillin-resistant *S*. *pseudintermedius* (MRSP). A wide range of MRSP prevalence rates have been reported in dogs, from as low as 2.6% in Norway [73], to as high as 34% in Brazil [74] and 40.5% in Canada [75]. The variations in the prevalence values may be due to the sites sampled as well as the methodology used to isolate the staphylococci and determine methicillin resistance. However, it is clear that MRSP is an increasing problem on a global scale, with two major strains ST 68 and ST 71 in circulation worldwide [76]. These data indicate that there is an urgent need for further investigations on the prevalence of MRSP in dogs as it may represent a public health issue.

Multidrug resistance was highest in CoPS (25.4%), lower values of 15.2% for CoNS and 10.3% for CoVS were also observed. The main pattern of resistance for all groups was the penicillin-tetracycline-trimethoprim/sulfamethoxazole combination. This is not surprising as penicillin, tetracycline and trimethoprim/sulfmethoxazole are common antimicrobials in Trinidad. Antimicrobial drugs can be purchased over-the-counter in Trinidad [77]. The frequent usage of these antimicrobials may contribute to selection pressures in the *Staphylococcus* spp. population.

## Conclusions

This study is the first to describe the distribution and antimicrobial susceptibility of staphylococci present on dogs and their owners in Trinidad. Striking is the high frequency of isolation of the SIG on humans, indicating a potential transfer of those strains between dogs and owners. Although, no MRSA were isolated, oxacillin-resistant SIG were detected in both dog and human isolates. MRCoNS were also detected and these may represent another reservoir for methicillin resistance. Further molecular studies are necessary to unambiguously demonstrate the potential exchange of staphylococci between dogs and their owners.

## Supporting information

S1 Table Clinical breakpoints, dosages, and species considerations for the various antimicrobial agents. (DOCX)

S2 Table Frequency of isolation of *Staphylococcus* spp. from the various sample sites on humans and animals. (XLSX)

S3 Table Frequency of isolation of antimicrobial resistant *Staphylococcus* spp. from dogs and humans. (XLSX)

S4 Table Prevalence values of antimicrobial resistant staphylococci on humans and dogs. (XLSX)

S1 Fig Distribution of isolates cultured from humans and dogs. (TIF)

S2 Fig Prevalence (%) of *Staphylococcus* spp. cultured from humans and dogs. (TIF)

S3 Fig Prevalence (%) of antimicrobial resistant CoPS in humans (n = 112) and animals (n = 112). (TIF)

S4 Fig Prevalence (%) of antimicrobial resistant CoNS+CoVS group in humans (n = 112) and animals (n = 112). (TIF)
