# Supplementary material for: Determination of the frequency, species distribution and antimicrobial resistance of staphylococci isolated from dogs and their owners in Trinidad
Source: PLoS One. 2021 Jul 2;16(7):e0254048. doi: 10.1371/journal.pone.0254048 (PMC8253405; doi:10.1371/journal.pone.0254048)
Supplement: S1 Table — (DOCX) [file pone.0254048.s001.docx]

**S1 Table.** Clinical breakpoints, dosages, and species considerations for the various antimicrobial agents extracted from the EUCAST breakpoint tables [40]

| Antimicrobial agent | Abbreviation | Dosage | Species  considerations | Zone diameter breakpoint (mm) | | |
| --- | --- | --- | --- | --- | --- | --- |
|  |  |  |  | S | I | R |
| Amikacin | AMK | 30 µg | *S. aureus* | 18 |  | 18 |
|  |  |  | CoNS | 22 |  | 22 |
| Penicillin G | PEN | 10U |  | 26 |  | 26 |
| Ciprofloxacin | CIP | 5 µg | *S. aureus* | 50 |  | 21 |
|  |  |  | CoNS | 50 |  | 24 |
| Tetracycline | TET | 30 µg |  | 22 | 20-21 | 19 |
| Chloramphenicol | CL | 30 µg |  | 18 |  | 18 |
| Trimethoprim/  Sulfamethoxazole | SXT | 25 μg |  | 17 |  | 14 |
| Cefoxitin (screening only) | FOX | 30 µg | *S. aureus* and CoNS other than *S. epidermidis* | 22 |  | 22 |
|  |  |  | *S. epidermidis* | 25 |  | 25 |
| Oxacillin (screening only) | OX | 1 µg | *S. pseudintermedius* | 20 |  | 20 |
