# Supplementary figures and images for: Determination of the frequency, species distribution and antimicrobial resistance of staphylococci isolated from dogs and their owners in Trinidad
Source: PLoS One. 2021 Jul 2;16(7):e0254048. doi: 10.1371/journal.pone.0254048 (PMC8253405; doi:10.1371/journal.pone.0254048)

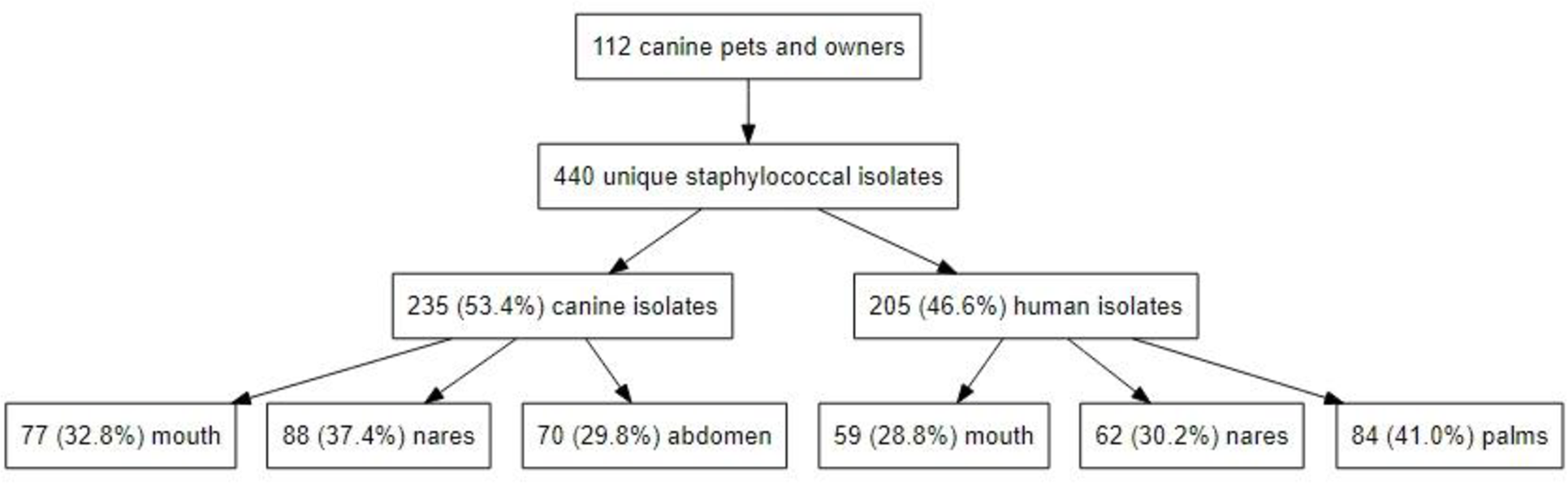

Supplement: S1 Fig — (TIF) [file pone.0254048.s005.tif]

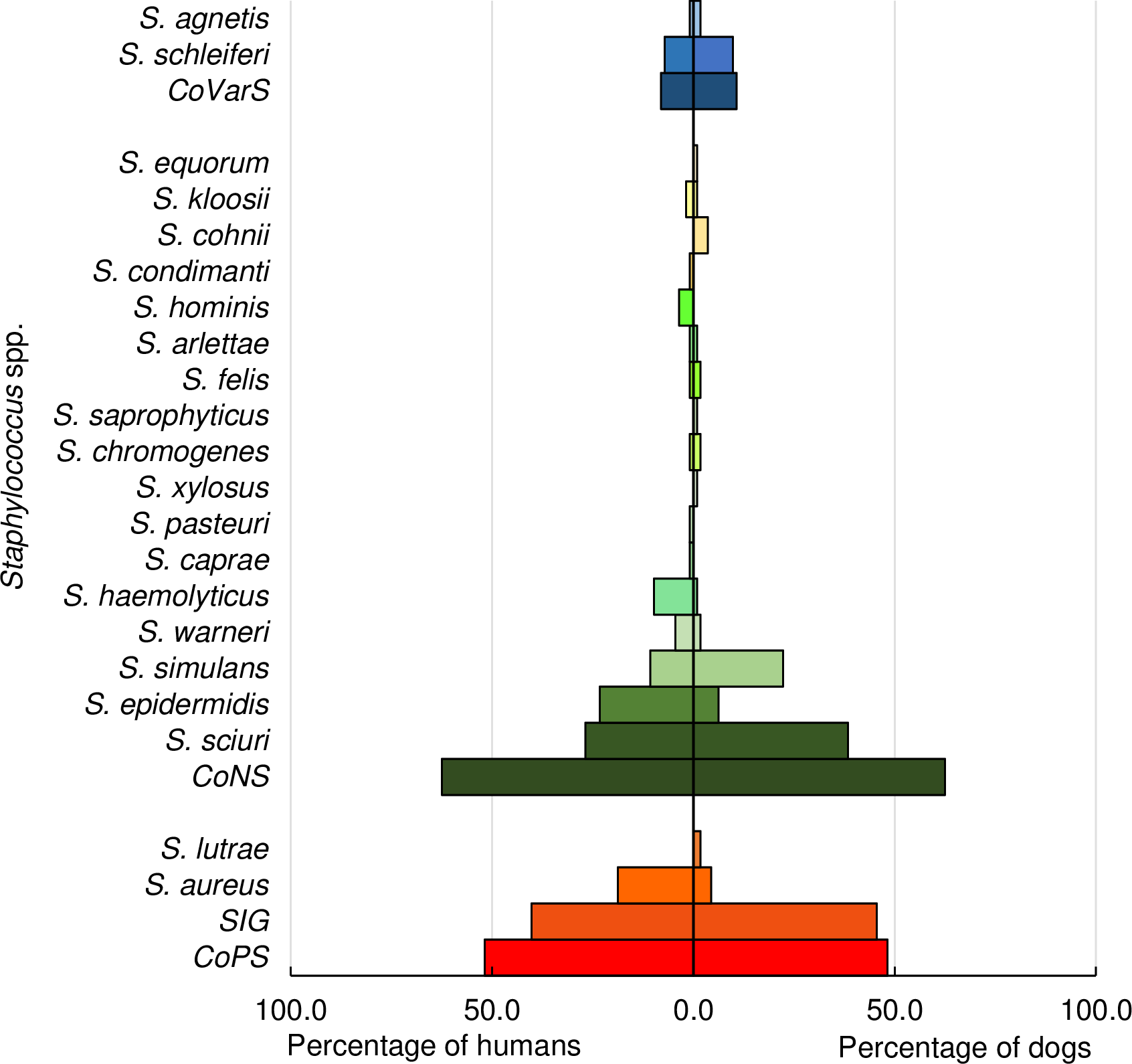

Supplement: S2 Fig — (TIF) [file pone.0254048.s006.tif]

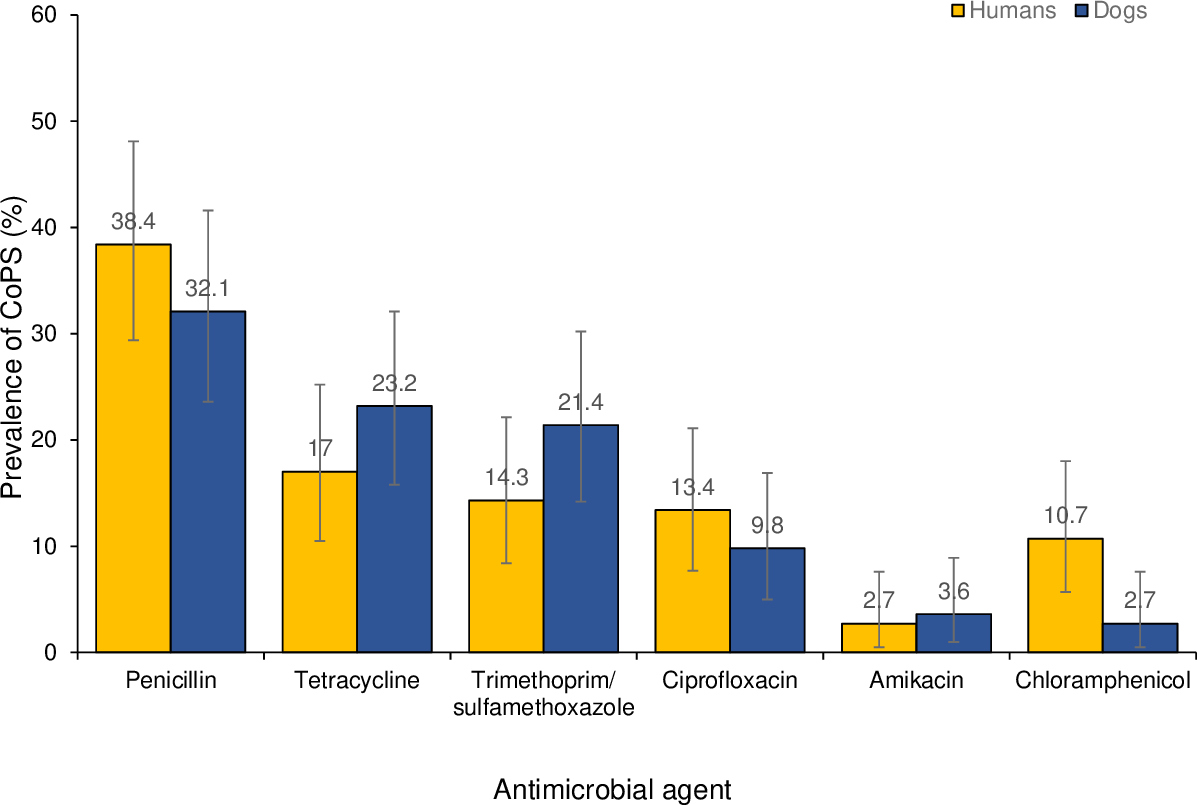

Supplement: S3 Fig — (TIF) [file pone.0254048.s007.tif]

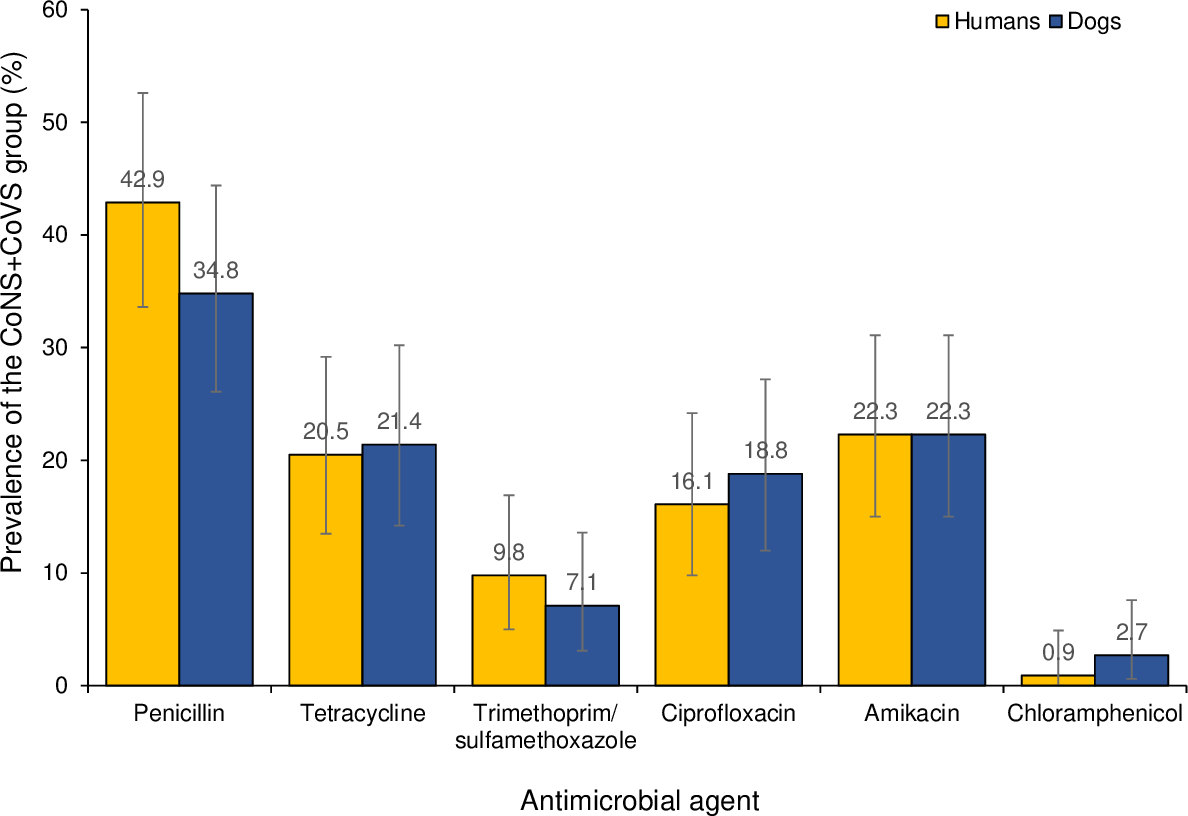

Supplement: S4 Fig — (TIF) [file pone.0254048.s008.tif]
